# Effect of urinary tract infection on the outcome of the allograft in patients with kidney transplantation

**DOI:** 10.1590/2175-8239-JBN-2024-0002en

**Published:** 2024-09-20

**Authors:** Rahul Sai Gangula, Mahesh Eshwarappa, R Rajashekar, Hamsa Reddy, Pooja Prakash Prabhu, Gireesh M Siddaiah, Gurudev Konana Chennabasappa, Lia Sara Anish, Yousuff Mohammad

**Affiliations:** 1 Ramaiah University of Applied Sciences (RUAS), M. S. Ramaiah Medical College, Department of Nephrology, Bengaluru, Karnataka, India. Ramaiah University of Applied Sciences (RUAS) M. S. Ramaiah Medical College Department of Nephrology Bengaluru Karnataka India

**Keywords:** Urinary tract Infection, Recurrent UTI, Non-Recurrent UTI, Transplantation

## Abstract

**Background:**

Urinary tract infections (UTIs) are the second most common cause of graft dysfunction, accounting for significant morbidity, and are associated with poor graft and patient survival. This study aimed to assess the association between post-renal transplant UTI and graft outcomes.

**Methods:**

We examined the effect of UTIs on graft outcomes in patients who underwent renal transplantation surgery between January 2010 and December 2022. The study population included 349 renal transplantations, of which 74 experienced 140 UTI events. Based on the number of UTI episodes, patients were categorized into three groups.

**Results:**

Of the 349 recipients, 275 (74.4%) had no UTI, 47 (18.8%) had non-recurrent UTIs (NR-UTIs), and 27 (6.8%) had recurrent UTIs (R-UTIs). Kaplan-Meier survival analysis showed that post-KT UTI status was a significant factor in graft survival, death-censored graft survival, and patient survival after a follow up of 5 years (log rank, P < 0.001). R-UTIs were associated with very poor graft survival and patient survival when compared with no UTI after a follow up of 5 years (hazard ratio [HR], 1.506; 95% confidence interval [CI], 1.233–1.840; P < 0.001 & HR, 1.529; 95% CI, 1.227–1.905; P = 0.001). R-UTIs were more likely to be associated with multi-drug resistant Gram-negative organisms (Klebsiella pneumonia or Escherichia coli) with resistance to nitrofurantoin (RR, 2.753; 95% CI, 1.257–6.032; P = 0.01) and carbapenem (RR, 2.064; 95% CI, 0.988–4.314; P = 0.05).

**Conclusion:**

Compared to no UTI, R-UTIs were associated to worse graft and patient outcomes after a follow-up of 5 years, whereas NR-UTIs were associated with poor graft and patient outcomes in the long term.

## Introduction

End-stage renal disease is the terminal stage of chronic kidney disease, in which the kidneys can no longer support the body’s needs^1^. Although various modalities of renal replacement therapies are available, kidney transplantation ensures a maximum life span with the best quality of life and is the most cost-effective^1^. Hence, kidney transplantation is currently considered the best modality for renal replacement therapy^1^.

After cardiovascular disease, infections are the second most common cause of mortality in kidney transplant recipients^1^. Urinary tract infections (UTIs) are among the most common infections after kidney transplantation and can lead to graft dysfunction and compromise the function of the transplanted kidney^1,2^. Although UTIs may occur at any time after renal transplantation, they are most common in the first year post-transplantation and may lead to sepsis, acute cellular rejection, impaired allograft function, graft loss, and patient death^1,2,3^. Hence, most centers prescribe at least 3–6 months of anti-microbial prophylaxis after kidney transplantation, although regimens may vary^1–4^. Despite routine administration of antimicrobial prophylaxis during the initial post-transplant period, it accounts for significant morbidity and mortality in transplant recipients^4^.

The incidence of UTI in kidney transplant recipients is highly variable between studies (7% – 80%). This variation is due to differences in patient populations, study designs, and UTIs definitions. Some UTIs may be asymptomatic or cause only mild symptoms, whereas others may lead to severe complications that affect graft function. Approximately 19% of patients develop acute pyelonephritis in the first 2 years after kidney transplantation. Common risk factors include prolonged indwelling catheters and Double J stents after transplantation, premature discontinuation of antibiotics, short length of transplant ureter, absence of sphincter between transplant ureter and native bladder, and net high dose of immunosuppression immediately after transplant^5,6,7^. Risk factors for recurrence include older age, female donor, deceased donor, neurogenic bladder, history of preoperative UTI, and acute rejection episodes (treated with steroids or immunosuppressive regimens)^5–7^. Recurrent UTIs have been reported to occur in 4–72% of transplant recipients^8^.

Although most of the studies^8,9,10,11,12^ have shown an association between post-kidney transplant UTI and deterioration of graft function, the impact of UTI on long-term graft and patient outcomes is less clear and has divergent results. This study was organized with the following objectives to address these critical knowledge gaps:

1) To determine the association between post-kidney transplant UTI and graft outcomes.
2) To determine the association between post-kidney transplant UTI and patient survival.
3) To describe and compare the microbiological and antimicrobial resistance profiles of patients with non-recurrent UTIs (NR-UTI) and recurrent UTIs (R-UTI).

## Methods

### Study Design and Patient Population

This hospital-based, observational, cohort study was conducted in a tertiary care hospital in Bengaluru, Karnataka, India. The study was approved by the Institutional Ethics Committee (DRP/IFP1085/2023). This study included all the patients who underwent kidney transplantation at our institute between January 2010 and December 2022.

### Definitions and Grouping of UTI Events

UTI was defined as the presence of any of the clinical symptoms of fever, dysuria, burning micturition, abdominal or loin pain, foul-smelling urine, increased frequency of micturition, and a urine culture sample revealing single microorganism growth with > 10^5^ bacterial colony-forming units per mL.

All patients were categorized into 3 groups based on their UTI status after kidney transplantation (no UTI, non-recurrent UTI, and recurrent UTI). Recurrent UTI was defined as a patient who had 2 or more UTI episodes in any 6 months (or) 3 or more episodes in any 12 months during post-transplant follow-up. Non-recurrent UTI was defined as all patients with a history of UTI after kidney transplantation who were not classified into the recurrent UTI group. No UTI was defined as all the patients who had never experienced any episode of UTI during post-kidney transplant follow-up.

Antibiotic resistance was defined according to the Clinical and Laboratory Standards Institute (CLSI) guidelines for antimicrobial susceptibility testing. Multi-drug resistance was defined as non-susceptibility to at least one agent in three or more different antimicrobial categories.

Graft failure was defined as impaired functioning of the graft kidney in the recipient, requiring renal replacement therapy for more than 3 months.

Asymptomatic bacteriuria was defined as patients who had no symptoms suggestive of a UTI but urine culture had grown organisms with >10^5^ colony-forming units per mL.

Acute cystitis was defined as a urinary tract infection confined to the bladder in an otherwise healthy, premenopausal, non-pregnant female.

### Posttransplant Immunosuppression

All the patients were stratified according to need for induction based on dialysis vintage, HLA mismatches, blood group compatibility, recipient age, and donor age. All high-risk patients received ATG, whereas moderate-risk patients received basiliximab. The low-risk patients were not administered ATG or basiliximab. Additionally, all patients received parenteral steroids on the day of transplantation and 2 days post-transplantation. All transplant recipients underwent ‘Lich-Gregoir’ ureterovesical anastomosis.

All the patients received a triple-drug maintenance immunosuppressive regimen consisting of Tacrolimus, Mycophenolate Mofetil, and Prednisolone. Tacrolimus was started at 0.08–0.1 mg/kg/day and subsequently, the dose was adjusted according to Tacrolimus trough levels. Tacrolimus trough target level of 9–10 ng/mL up to 3 months post-transplant, a target of 7–9 ng/mL from 3 to 12 months post-transplant, and a target of 5–7 ng/mL thereafter were considered. Mycophenolate mofetil 500 mg was administered twice to thrice daily. Steroids were tapered to 10 mg 3 months post-transplantation and 10 mg was maintained until 12 months post-transplantation. Thereafter steroids were tapered to 5–7.5 mg per day, which was continued thereafter.

### Catheter and DJ Stent Policy

A double J-stent was inserted into all allograft recipients during transplantation as a standard procedure and was removed aseptically between 3 and 8 weeks after transplantation. All transplant recipients were placed on Foley catheters during the kidney transplantation. They were usually removed between postoperative days 5 and 9 unless the patient had a neurogenic bladder or any other indication where prolonged Foley catheterization was recommended.

### Post-Transplant Chemoprophylaxis

CMV seronegative patients who received a kidney from a CMV seropositive donor and deceased donor kidney transplant recipients were administered valganciclovir during the first 6 months post-transplantation. Additionally, trimethoprim-sulfamethoxazole for *Pneumocystis jiroveci* was administered during the first 3 months post-transplant if it was tolerated.

### Asymptomatic Bacteriuria

If a patient was found to have asymptomatic bacteriuria in the initial 2 months post-transplant, urine culture was repeated. If the patient had two consecutive urine cultures that yielded >10^5^ colony-forming units of the same pathogen, they were treated with antibiotics for 5 days according to culture sensitivity and were considered as UTI in the analysis. Asymptomatic bacteriuria beyond 2 months post-transplantation was not treated with antibiotics and not considered in the analysis.

### Treatment of UTI

All UTI events without sepsis were treated empirically with third-generation cephalosporins. Events of urosepsis were treated empirically with carbapenems. Following urine culture reports, the prescription was adjusted and appropriate antibiotics were administered for an optimal duration (i.e. 14–21 days). All patients with recurrent UTI underwent clinical, laboratory, and imaging workups to identify the cause and were treated accordingly.

### Post-UTI Antibiotic Prophylaxis

Transplant recipients with recurrent UTIs were treated appropriately and antibiotic prophylaxis was administered for 6 weeks to 3 months.

### Diagnosis of Rejection, BK Virus Nephropathy

Rejection episodes were diagnosed using graft kidney biopsy, graded according to the Banff classification^13^, and treated accordingly. BKV PCR screening was performed periodically in all transplant recipients. BK viral nephropathy was confirmed using allograft biopsy tissue SV40 staining.

### Outcomes

The primary outcome was the overall graft survival in patients with UTI. The secondary outcomes included yearly graft function, death-censored graft survival, patient survival, and association between UTI status and antibiotic resistance. Patients who had functioning grafts on the day of death were censored to analyze death-censored graft survival.

### Data Collection

Data were collected using a predesigned proforma and subsequently entered into Microsoft Office Professional Plus Excel 2016, version 16.0 (Microsoft Corp, Redmond, USA). To avoid any possible error, the data entry was cross-checked at two levels (entry into the proforma and entry from the proforma to the Excel sheet) by two independent observers.

### Statistical Analysis

Descriptive statistics were performed for categorical and continuous variables. The Shapiro-Wilk test of normality was used to check data distribution. A P value of < 0.05 in the Shapiro-Wilk test of normality was considered significant and the distribution was classified as a non-Gaussian distribution. Quantitative variables with a Gaussian distribution were summarized as means and standard deviations. The quantitative variables, which had a skewed distribution, were summarized as medians and interquartile ranges. All patients were categorized into three groups (no UTI, non-recurrent UTI, recurrent UTI) based on post-kidney transplant UTI. Kidney transplant recipient, donor, and transplantation characteristics were compared using the chi-square test for categorical data and one-way analysis of variance (ANOVA), or the Kruskal-Wallis H test for continuous variables. Patient, graft, and death-censored graft survival outcomes were computed and compared using Kaplan-Meier survival analyses (log-rank test, P < 0.05 is considered significant) and univariate Cox regression. The mean and median survival times with 95% confidence intervals were computed for the graft and patient outcomes.

Cox proportional hazard models were derived using variables selected using a backward stepwise approach. Variables associated with graft failure (P < 0.20) were considered for inclusion in the model. Only the variables that significantly altered the relationship between post-transplant UTI status and outcome, resulting in a ≥ 10% change in the associated HR, were included in the final multivariate model. UTI was considered a time-dependent variable. The unadjusted risk ratios for antibiotic resistance were compared with the UTI status for Gram-negative species at each episode and at the patient level, P value of <0.05 was considered statistically significant. All statistical analyses were performed using the Statistical Package for the Social Sciences (SPSS) Statistics, version 25 (IBM Co., Armonk, NY, USA), and DATA *tab* Statistics calculator (Graz, Austria).

## Results

**Figure 1:**
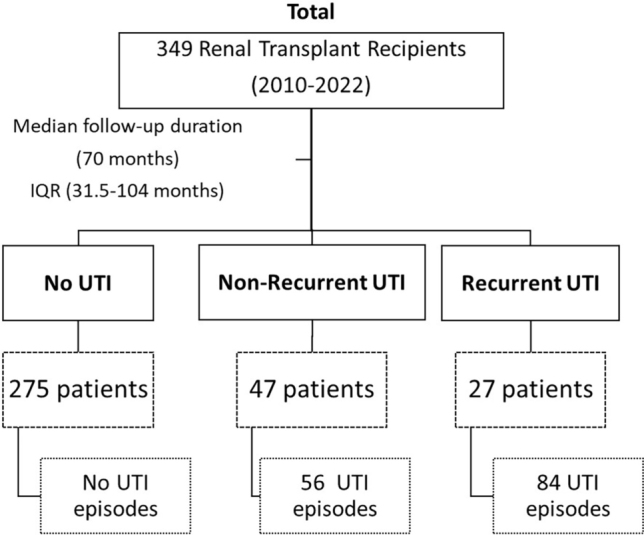
Baseline flow chart of study design.

A total of 349 patients underwent kidney transplantation during the specified period (2010-2022) and all patients were included in the final analysis (Figure 1). The median follow-up duration was 70 months (IQR 31.5-104 months). A total of 287 patients were male and 62 were female (Figure S1). The mean age of the transplant recipients was 37.49 years with a standard deviation of 11.92 years. During the study period, 74 patients experienced 140 UTI episodes. Of these, 47 (13%) patients experienced NR-UTIs, whereas 27 (8%) patients had R-UTIs. A total of 56 (40%) and 84 (60%) UTI episodes were noted in the patients with NR-UTI and with R-UTI, respectively. The other baseline characteristics are summarized in Table 1.

**Table 1:** Comparison of recipient, donor, and transplantation-related characteristics based on post-kidney transplant UTI status

Characteristics		Total (n = 349)	No UTI (n = 275)	Non-Recurrent UTI (n= 47)	Recurrent UTI (n = 27)	P value
**Recipient characteristics**						
**Age**	**Mean**	37.49	36.29	40.83	42.74	**0.003**
	**Standard Deviation**	11.92	11.66	12.03	12.16
**Sex**	**Male**	287 (82.2%)	228 (82.9%)	36 (76.6%)	23 (85.2%)	0.530
	**Female**	62 (17.8%)	47 (17.9%)	11 (23.4%)	4 (14.8%)
**Basic Disease**	**CGN**	201 (57.6%)	171 (62.1%)	21 (44.7%)	9 (33.3%)	**0.002**
	**DKD**	66 (18.9%)	49 (17.8%)	11 (23.4%)	6 (22.2%)	0.601
	**CIN**	53 (15.2%	36 (13.1%)	12 (25.5%)	5 (18.5%)	0.079
	**Others**	10 (2.9%)	7 (2.5%)	0 (0.0%)	3 (11.1%)	**0.017**
	**Unknown**	19 (5.4%)	12 (4.4%)	3 (6.4%)	4 (14.8%)	0.070
**Diabetes Mellitus**		59 (16.9%)	45 (16.4%)	8 (17.0%)	6 (22.2%)	0.742
**Hypertension**		281 (80.5%)	216 (78.5%)	38 (80.1%)	21 (77.8%)	0.668
**Ischemic Heart Disease**		29 (8.3%)	27 (9.8%)	1 (2.1%)	1 (3.7%)	0.141
**Cerebrovascular Disease**		9 (2.6%)	4 (14.5%)	2 (4.2%)	3 (11.1%)	**0.008**
**Seizure Disorder**		13 (3.7%)	9 (3.3%)	4 (8.5%)	0 (0.0%)	0.123
**Hypothyroidism**		39 (11.2%)	32 (11.6%)	5 (10.6%)	2 (7.4%)	0.796
**Pre-Transplant UTI**		12 (3.4%)	8 (2.9%)	2 (4.2%)	2 (7.4%)	0.450
**Donor Characteristics**						
**Age**	**Mean**	45.26	44.77	46.15	46.30	0.638
	**Standard Deviation**	10.80	11.46	11.54	12.29
**Sex**	**Male**	94 (26.9%)	68 (24.7%)	17 (36.2%)	9 (33.3%)	0.194
	**Female**	255 (73.1%)	209 (75.3%)	28 (63.8%)	18 (66.7%)
**Deceased donor**		46 (13.2%)	31 (11.3%)	10 (21.3%)	5 (18.5%)	0.086
**Marginal living donor**		44 (12.6%)	38 (13.8%)	4 (8.5%)	2 (7.4%)	**<0.001**
**Transplantation characteristics**						
**HLA > 3 mismatches**		145 (41.5%)	111 (40.4%)	20 (42.6%)	14 (51.9%)	0.509
**Second Transplant**		8 (2.3%)	5 (1.8%)	2 (4.3%)	1 (2.1%)	0.518
**TMP-SMX prophylaxis**		297 (85.1%)	241 (87.6%)	36 (76.6%)	20 (74.1%)	**0.036**

### Recipient Characteristics

Recipients with a higher mean age (Figure S2) were more likely to experience NR-UTIs and R-UTI than patients with no UTI events (p = 0.039, p = 0.019 respectively), and there was no statistically significant difference between NR-UTIs and R-UTI (p = 0.779). Recipients with R-UTIs were more likely to have cerebrovascular disease than patients with no UTI events (P = 0.007). However, there was no statistically significant difference between NR-UTIs and patients with No UTI events (P = 0.498). Kidney transplant recipients with chronic glomerulonephritis and CAKUT as native kidney disease (Table 1, Figure S3) were associated with R-UTIs (vs No UTI, P = 0.019; vs No UTI and NR-UTI, P = 0.009 and P = 0.029 respectively).

### Donor Characteristics

Kidney transplant recipients with marginal living donors were associated with R-UTI and NR-UTIs when compared with patients with no episodes of UTI (P = 0.007, p = 0.003 respectively).

#### Transplant Characteristics

Transplant recipients with post-transplant diabetes mellitus (PTDM) were associated with R-UTIs compared with patients with NR-UTIs and patients with No UTI episodes (P = 0.005, P = 0.002 respectively). Patients who did not receive trimethoprim-sulfamethoxazole prophylaxis developed NR-UTIs (vs No UTI, P = 0.049). Kidney transplant recipients with delayed graft function had non-recurrent UTIs (vs. No UTI, P = 0.038) but the difference was not statistically significant for recurrent UTIs (vs. No UTIs, P = 0.459). Patients with persistent DJ stents for > 4 weeks had R-UTI (vs No UTI, P < 0.001). Transplant recipients who had not received any induction agents were associated with R-UTI (P = 0.019) compared with patients who did not experience any urinary tract infections.

#### Number of UTI Episodes in Each Patient

A total of 41 (55%) patients experienced only 1 UTI event, 16 (22%) patients had 2 UTI events, 10 (13%) patients had a sum of 3 UTI events, and 7 (10%) patients had more than 3 UTI events (Figure S4).

#### Median Time for UTI Event

The median time from transplantation to initial UTI in NR-UTI and R-UTI was 41 days (IQR 16.0 – 624.5 days) and 26 days (IQR 19.5–56.5 days) respectively (Figure S5).

#### Graft and Patient Outcomes at the 5-year Follow-up

**Figure 2:**
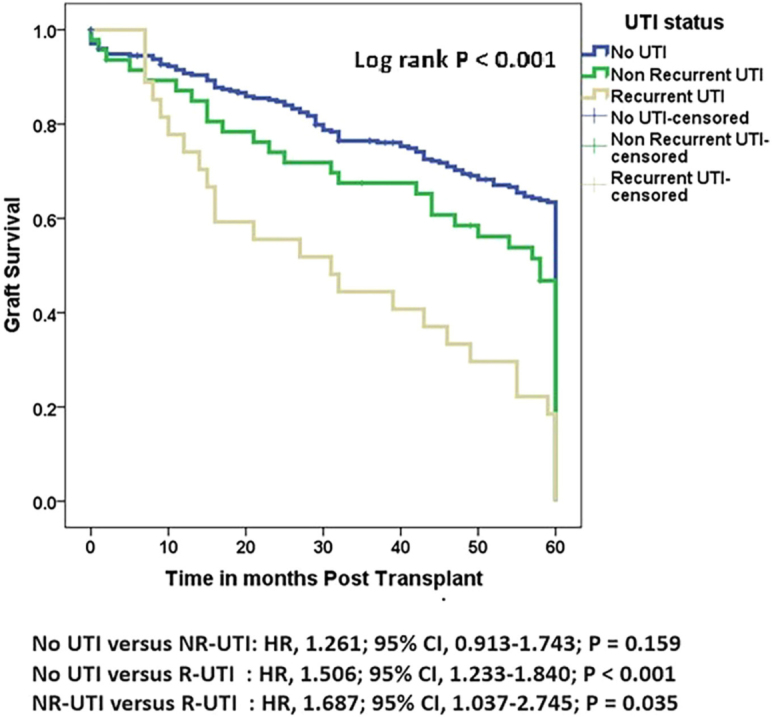
Comparison of graft survival by UTI status following kidney transplantation (followed up for 5 years). Results are shown for recipients with no UTI, non-recurrent UTI, and recurrent UTI.

**Figure 3:**
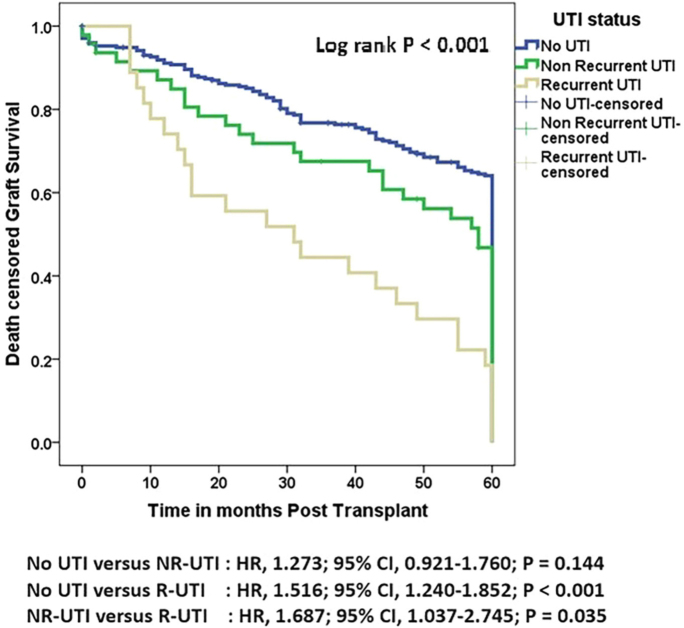
Comparison of death-censored graft survival by UTI status following kidney transplantation (followed up for 5 years). Results are shown for recipients with no UTI, non-recurrent UTI, and recurrent UTI.

**Figure 4:**
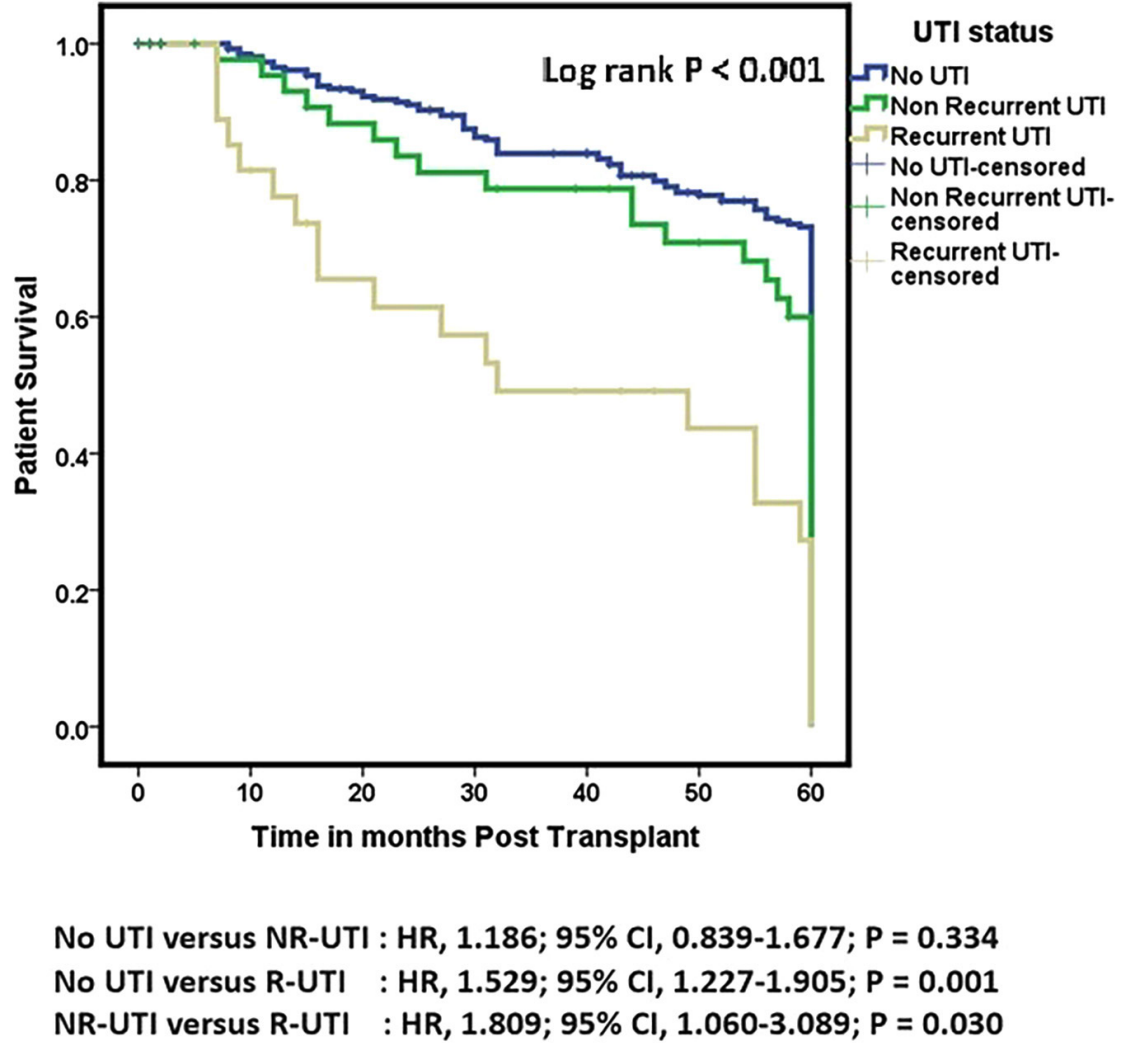
Comparison of patient survival by UTI status following kidney transplantation (followed up for 5 years). Results are shown for recipients with no UTI, non-recurrent UTI, and recurrent UTI.

Kaplan-Meier survival analysis showed that post-KT UTI status was significantly different in graft survival, death-censored graft survival, and patient survival after a follow up of 5 years. (Figures 2–4, Tables S1–S3).

After adjusting for confounding factors in the multivariable Cox regression analysis, NR-UTI [HR 2.318; p = 0.001) was associated with a higher risk of graft failure than no UTI or R-UTI (Table 2 and 3).

**Table 2:** Cox regression model of recipient factors associated with overall graft failure among kidney transplant recipients

Parameter		Adjusted hazard ratio	95% Confidence interval	P value
**UTI status**	**No UTI**	Comparator	–	–
	**Non-Recurrent UTI**	2.318	1.414–3.800	**0.001**
	**Recurrent UTI**	1.528	0.657–3.551	0.325
**Recipient Age**		1.026	1.007–1.046	**0.008**
**Recipient Gender**		0.657	0.403–1.070	0.091
**Hypertension**		1.182	0.956–1.343	0.065
**Diabetes**		1.304	0.969–1.756	0.080
**Post-transplant Diabetes**		1.912	1.423–2.257	**0.007**
**Previous transplant**		1.113	0.550–2.254	0.766

**Table 3:** Cox regression model of transplant and donor factors associated with overall graft failure among kidney transplant recipient

Parameter		Adjusted hazard ratio	95% Confidence interval	P value
**UTI status**	**No UTI**	Comparator	–	–
	**Non-Recurrent UTI**	2.318	1.414–3.800	**0.001**
	**Recurrent UTI**	1.528	0.657–3.551	0.325
**Delayed Graft Function**		2.202	1.614–3.003	**<0.001**
**HLA > 3 mismatch**		1.307	1.041–1.641	**0.021**
**Induction agent**	**ATG**	Comparator	–	–
	**Basiliximab**	2.467	1.437–4.236	0.001
	**Nil**	1.138	0.724–1.790	0.003
**Rejection/Transplant Glomerulopathy**		8.840	5.472–14.281	**<0.001**
**Recurrence of Basic disease**		5.452	3.117–9.537	**<0.001**
**BK virus nephropathy**		1.496	0.986–1.984	**0.032**
**Donor Gender**		1.004	0.615–1.637	0.989
**Marginal Living Donor**		1.598	1.262–2.023	**<0.001**
**Deceased Donor**		1.961	1.420–2.709	**<0.001**

A total of 34 (10%) patients had graft loss during the observation period, of whom 26 (9%) had no UTI and 8 (17%) had non-recurrent UTI. The median overall graft survival time was 72 months (95% CI 64.654–79.346). However, the median overall graft survival times for the No-UTI, NR-UTI, and R-UTI groups were 84 months (95% CI 73.763–94.237), 60 months (95% CI 48.817–71.183), and 36 months (95% CI 1.271–70.729), respectively (Table S4 and S5).

A total of 56 (16%) patients died during the observation period, of whom 37 (13%) had no UTI, 13 (27%) had non-recurrent UTI, and 6 (22%) had recurrent UTI. The median patient survival time for the NR-UTI group was 118 months (95% CI 90.932–145.068) (Table S6).

#### Microbiological Profile and Resistance Patterns

A total of 140 UTI events were noted in 74 patients during the observation period, of which 133 (95%) were due to Gram-negative bacteria and 7 (5%) were due to Gram-positive bacteria (Table 4). The most common Gram-negative bacteria were *Klebsiella pneumoniae* (47%) and *Escherichia coli* (41%) whereas the most common Gram-positive bacteria were Enterococcus species (Figure S6). Most of the organisms were multidrug resistant, accounting for 38 (73.07%) and 58 (71.6%) events in non-recurrent and recurrent UTIs, respectively (Table S7).

**Table 4:** Patient-level analysis of antimicrobial resistance patterns for gram-negative organisms causing non-recurrent and recurrent post-kidney transplant UTIs

Antibiotic-Resistant	Non-Recurrent UTI (52)	Recurrent UTI (81)	RR	95% CI	P-value
**3^rd^ Generation Cephalosporin**	43 (87.8)	68 (85)	0.791	0.276–2.264	0.081
**Extended-spectrum Beta-lactam**	9 (22.5)	11 (16.0)	0.709	0.270–1.856	0.486
**4^th^ Generation Cephalosporin**	40 (81.6)	54 (67.5)	0.467	0.197–1.106	0.664
**Aminoglycosides**	32 (64)	43 (53.7)	0.654	0.316–1.351	0.253
**Fluoroquinolones**	45 (90)	73 (91.2)	1.159	0.347–3.871	0.812
**Nitrofurantoin**	25 (54.3)	59 (76.6)	2.753	1.257–6.032	**0.010**
**Carbapenem**	26 (53.0)	56 (70.0)	2.064	0.988–4.314	**0.049**
**Cotrimoxazole**	39 (81.2)	63 (78.7)	0.855	0.347–2.108	0.736

## Discussion

Although there are no unique criteria for distinguishing early from late UTIs, UTIs occurring within a year after transplantation have been termed early UTIs in numerous studies^1–7^. The timing of UTI episodes post-transplantation is critical, as studies have indicated that early UTI is a risk factor for the development of sepsis and allograft rejection^4–7^. Similarly, late recurrent UTI increases the likelihood of allograft dysfunction and graft loss^8^. However, these effects have not been consistent across all investigations^4,8,10^. Recent data suggest that even a single episode of UTI can compromise the long-term allograft performance in kidney transplant recipients^4,12^.

The processes of allograft damage by pathogens targeting the urinary tract are linked to the UTI-associated inflammatory response to bacterial invasion, which is caused by immunological dysregulation and both local and systemic activation of cytokines such as TNF-α, IL-1, IL-6, and IL-8^7^. Furthermore, cytokine release is thought to play a role in the etiology of allograft rejection by hastening the exposure of allograft tissues to HLAs, resulting in the activation of leukocytes and vascular endothelial cells^7^. In certain situations, the development of acute pyelonephritis can potentially lead to chronic allograft failure due to direct kidney injury^12^.

Infection with virulent Gram-negative uropathogenic organisms with specialized structures such as P fimbriae (pyelonephritis-associated pili) is strongly linked to acute allograft dysfunction^7^. Repeated attacks deplete the regenerative capacity of the graft tissue and promote irreversible fibrosis^7^. As a result, in transplant recipients with late recurrent UTI, allograft damage can result in the formation of numerous localized scarring abnormalities that can be observed by Technetium Tc 99m Di-mercapto succinic acid single photon emission computed tomography (99mTc-DMSA SPECT)^7^.

The present study is unique in that it considered all patients who underwent kidney transplantation during the study period and none were excluded. This study aimed to evaluate and determine the association between UTI after kidney transplantation and graft and patient outcomes. In concordance with other studies^4,8^, most cases of UTI events (both R-UTI and NR-UTI) occurred within a year post-transplant. This might be due to the higher net dose of immunosuppression immediately after transplantation.

According to the findings of the current study, older recipients, recipients with marginal living donors, and transplant recipients who had developed DGF or PTDM were more likely to develop UTI. This could be explained by an altered immunological balance, persistently high blood glucose levels, and impaired mucosal barrier function during these special conditions.

A novel finding of the present study was that NR-UTI was associated with a greater risk of graft failure and inferior patient and graft outcomes. This contradicts most previous studies^4,8^ that showed that R-UTIs are generally associated with worse patient and graft outcomes. Although the current study demonstrated an increase in mortality in R-UTI patients, it was not statistically significant when compared with those with No UTI. A possible explanation for the lower graft survival among NR-UTI compared to R-UTI could be that patients with R-UTI received empirical treatment with carbapenems, prolonged antibiotic prophylaxis, close monitoring and follow-up. This could be due to the intensive approach to root cause analysis of recurrent UTI and early treatment strategies. Some patients had reflux to the native kidney and underwent surgical procedures (1 patient underwent native kidney nephrectomy and 3 transplant recipients had dextranomer/hyaluronic acid bulking agent injected at the vesicoureteral junction). However, the discrepancy may be attributable to the shorter follow-up time in these instances, as most patients with R-UTIs had undergone transplant recently (within the last 5 years). This is evident when a survival analysis is conducted with only a 5-year follow-up.

India is a large and diverse country with a wide range of infectious diseases due to population density, inadequate sanitation, economic inequality, access to healthcare, and varying levels of vaccine coverage. Due to the associated increased morbidity and mortality, anti-microbial resistance has become a serious health problem. Similar to previous studies, Gram-negative organisms accounted for the majority of UTIs, with *Klebsiella pneumoniae* and *Escherichia coli* accounting for approximately 47% and 41%, respectively. The pathogens causing R-UTIs were analogous to those involved in NR-UTI, with no statistically significant differences observed between the two groups for any organism.

When analyzed at the patient and UTI event levels, both NR-UTI and R-UTIs were more likely to be caused by multidrug-resistant organisms. Resistance to carbapenems or nitrofurantoin is associated with the development of recurrent UTIs, leading to treatment challenges. This is likely due to the ineffective practice of antibiotic stewardship. This is leading to an increase in multidrug-resistant pathogens, particularly nitrofurantoin and carbapenem resistance, as these antibiotics are currently regularly used to treat complicated UTIs.

The divergent outcomes observed in the current study could be explained by the early treatment techniques and the root cause analysis approach used in the evaluation of R-UTIs. The current study’s significant drawback is that the majority of R-UTI patients underwent recent transplants; therefore, their long-term graft (>5 years) outcome could not be studied. However, it is planned to follow all these recently transplanted patients in the long term and investigate their transplant outcomes.

The inclusion of all kidney transplant recipients throughout the research period and the extensive follow-up period were strengths of this study. An independent risk factor evaluation of UTI-related graft dysfunction was performed after the influence of confounding variables was assessed using a multivariable Cox proportional regression analysis. To quantify the independent risk of confounding factors, proportional hazards, and hazard ratios were obtained. Asymptomatic bacteriuria occurring in the first 2 months after transplantation was confirmed by repeat culture and treated if the same organism was isolated^14^.

However, this study had several limitations. We did not assess the overall prevalence of asymptomatic bacteriuria and its relationship with the emergence of UTI following kidney transplantation. As this was a single-center study, it is not possible to extrapolate the findings to the entire target population. In the current study, female patients were underrepresented as transplant recipients and donors were strongly overrepresented. This could be due to the fact that donors in India are predominantly mothers or spouses^15^. Therefore, the results cannot be generalized to other transplant centers with a balanced sex distribution of recipients and donors. This study did not explore the relationship between the different types and durations of therapy and their impact on outcomes. The R-UTI group in the current study underwent transplantation more recently and did not have a long follow-up period. This is one of the shortcomings of the study that could be addressed by a longer follow-up of these transplant recipients.

In summary, we found that the NR-UTI group had consistently poorer graft performance, graft survival, and patient outcomes than the no-UTI group. The most likely time for UTIs after kidney transplantation is within one year of transplantation, and the most common cause is multidrug-resistant Gram-negative bacteria. Additionally, certain factors were found to be independently associated with graft failure, including male recipients, post-transplant diabetes mellitus, graft glomerulopathy, recurrence of underlying disease/de-novo glomerular disease, marginal living donors, deceased donors, delayed graft function, HLA > 3 mismatches, BK virus nephropathy, rejection episodes, no induction, and basiliximab as an induction agent.

## Conclusions

Compared to no UTI, R-UTIs were associated with poorer graft and patient outcomes after a 5-year follow-up, while NR-UTIs were associated with poorer graft and patient outcomes in the long term. Given the scarcity of available treatments, it is crucial to combat antimicrobial multi-drug resistance, particularly carbapenem resistance. For people who have had a kidney transplantation, even a single UTI can result in poor long-term graft and patient outcomes.

## Supplementary Material

The following online material is available for this article:

Figure S1 – Sankey chart showing the gender of kidney transplant recipients and donors.

Figure S2 – Box and whisker plot with data points representing the age of kidney transplant recipients and donors.

Figure S3 – Sankey chart showing the native kidney disease of kidney transplant recipients.

Figure S4 – Sankey chart showing the total number of UTI episodes in each patient.

Figure S5 – Vertical raincloud plot showing the duration of the first episode of UTI post-kidney transplant in non-recurrent and recurrent UTI groups.

Figure S6 – Sankey chart showing the antimicrobial profile of Gram-negative organisms causing post-renal transplant UTI.

Table S1 – Mean and median survival time of graft survival at a 5 years follow-up.

Table S2 – Mean and median survival time of death-censored graft survival at a 5 years follow-up.

Table S3 – Mean and median survival time of patient survival at a 5 years follow-up.

Table S4 – Mean and median survival time of overall graft survival.

Table S5 – Mean and median survival time of overall death-censored graft survival.

Table S6 – Mean and median survival time of overall patient survival.

Table S7 – Patient analysis of Gram-negative organisms with antibiotic resistance causing non-recurrent and recurrent post-kidney transplant UTIs (on case-by-case basis).
