# Supplementary material for: Effect of urinary tract infection on the outcome of the allograft in patients with kidney transplantation
Source: J Bras Nefrol. 2024 Sep 20;46(4):e20240002. doi: 10.1590/2175-8239-JBN-2024-0002en (PMC11420934; doi:10.1590/2175-8239-JBN-2024-0002en)
Supplement: Supplementary file 6 [file 2175-8239-jbn-46-4-e20240002-suppl7.pdf]

Braz. J. Nephrol. (J. Bras. Nefrol.)

https://doi.org/10.1590/2175-8239-JBN-2024-0002en

Supplementary Material to "Effect of urinary tract infection on the outcome of the allograft in patients with kidney transplantation"

Table S1. Mean and median graft survival at 5 years follow up.

UTI status	Mean				Median			
	Estimate	Standard Error	95% Confidence Interval		Estimate	Standard Error	95% Confidence Interval	
			Lower Bound	Upper Bound			Lower Bound	Upper Bound
**No UTI**	48.461	1.149	46.209	50.713	60.000	0.000	-	-
**Non-Recurrent UTI**	43.284	3.159	37.092	49.476	58.000	1.333	55.388	60.612
**Recurrent UTI**	32.519	4.046	24.589	40.448	31.000	9.520	12.341	49.659
**Overall**	46.477	1.074	44.372	48.581	60.000	0.000	-	-
