# Supplementary material for: Effect of urinary tract infection on the outcome of the allograft in patients with kidney transplantation
Source: J Bras Nefrol. 2024 Sep 20;46(4):e20240002. doi: 10.1590/2175-8239-JBN-2024-0002en (PMC11420934; doi:10.1590/2175-8239-JBN-2024-0002en)
Supplement: Supplementary file 7 [file 2175-8239-jbn-46-4-e20240002-suppl9.pdf]

*Braz. J. Nephrol. (J. Bras. Nefrol.)*

https://doi.org/10.1590/2175-8239-JBN-2024-0002en

# Supplementary Material to "Effect of urinary tract infection on the outcome of the allograft in patients with kidney transplantation"

**Table S3.** Mean and median patient survival at 5 years follow up.

| UTI status | Mean | | | | Median | | | |
|---|---|---|---|---|---|---|---|---|
| | Estimate | Standard Error | 95% Confidence Interval | | Estimate | Standard Error | 95% Confidence Interval | |
| | | | Lower Bound | Upper Bound | | | Lower Bound | Upper Bound |
| **No UTI** | 52.643 | 0.915 | 50.850 | 54.436 | 60.000 | 0.000 | - | - |
| **Non-Recurrent UTI** | 49.530 | 2.667 | 44.303 | 54.758 | 60.000 | 0.000 | - | - |
| **Recurrent UTI** | 36.560 | 4.403 | 27.930 | 45.191 | 32.000 | 16.077 | 0.488 | 63.512 |
| **Overall** | 50.979 | 0.903 | 49.208 | 52.749 | 60.000 | 0.000 | - | - |
