# Supplementary material for: Effect of urinary tract infection on the outcome of the allograft in patients with kidney transplantation
Source: J Bras Nefrol. 2024 Sep 20;46(4):e20240002. doi: 10.1590/2175-8239-JBN-2024-0002en (PMC11420934; doi:10.1590/2175-8239-JBN-2024-0002en)
Supplement: Supplementary file 8 [file 2175-8239-jbn-46-4-e20240002-suppl10.pdf]

Braz. J. Nephrol. (J. Bras. Nefrol.)

https://doi.org/10.1590/2175-8239-JBN-2024-0002en

Supplementary Material to “Effect of urinary tract infection on the outcome of the allograft in patients with kidney transplantation”

Table S4. Mean and median over-all graft survival.

UTI status	Mean				Median			
	Estimate	Standard Error	95% Confidence Interval		Estimate	Standard Error	95% Confidence Interval	
			Lower bound	Upper Bound			Lower Bound	Upper Bound
**No UTI**	78.032	2.730	72.680	83.383	84.000	5.223	73.763	94.237
**Non-Recurrent UTI**	61.759	5.033	51.893	71.624	60.000	5.706	48.817	71.183
**Recurrent UTI**	42.918	6.324	30.523	55.313	36.000	17.719	1.271	70.729
**Overall**	73.734	2.419	68.993	78.476	72.000	3.748	64.654	79.346
