# Supplementary material for: Effect of urinary tract infection on the outcome of the allograft in patients with kidney transplantation
Source: J Bras Nefrol. 2024 Sep 20;46(4):e20240002. doi: 10.1590/2175-8239-JBN-2024-0002en (PMC11420934; doi:10.1590/2175-8239-JBN-2024-0002en)
Supplement: Supplementary file 9 [file 2175-8239-jbn-46-4-e20240002-suppl11.pdf]

Braz. J. Nephrol. (J. Bras. Nefrol.)

https://doi.org/10.1590/2175-8239-JBN-2024-0002en

Supplementary Material to "Effect of urinary tract infection on the outcome of the allograft in patients with kidney transplantation"

Table S5. Mean and median of death-censored graft survival.

UTI status	Mean				Median			
	Estimate	Standard Error	95% Confidence Interval		Estimate	Standard Error	95% Confidence Interval	
			Lower bound	Upper Bound			Lower Bound	Upper Bound
**No UTI**	122.086	3.872	114.497	129.675	-	-	-	-
**Non-Recurrent UTI**	73.336	6.520	60.556	86.116	80.000	7.333	65.627	94.373
**Recurrent UTI**	78.168	10.511	57.566	98.771	-	-	-	-
**Overall**	116.932	3.615	109.846	124.018	-	-	-	-
