# Supplementary material for: Effect of urinary tract infection on the outcome of the allograft in patients with kidney transplantation
Source: J Bras Nefrol. 2024 Sep 20;46(4):e20240002. doi: 10.1590/2175-8239-JBN-2024-0002en (PMC11420934; doi:10.1590/2175-8239-JBN-2024-0002en)
Supplement: Supplementary file 10 [file 2175-8239-jbn-46-4-e20240002-suppl12.pdf]

Braz. J. Nephrol. (J. Bras. Nefrol.)

https://doi.org/10.1590/2175-8239-JBN-2024-0002en

Supplementary Material to "Effect of urinary tract infection on the outcome of the allograft in patients with kidney transplantation"

Table S6. Mean and median patient survival.

UTI status	Mean				Median			
	Estimate	Standard Error	95% Confidence Interval		Estimate	Standard Error	95% Confidence Interval	
			Lower bound	Upper Bound			Lower Bound	Upper Bound
**No UTI**	137.514	3.272	131.101	143.928	-	-	-	-
**Non-Recurrent UTI**	96.168	7.748	80.982	111.353	118.000	13.810	90.932	145.068
**Recurrent UTI**	78.168	10.511	57.566	98.771	-	-	-	-
**Overall**	132.724	3.177	126.497	138.952	-	-	-	-
