# Supplementary material for: Effect of urinary tract infection on the outcome of the allograft in patients with kidney transplantation
Source: J Bras Nefrol. 2024 Sep 20;46(4):e20240002. doi: 10.1590/2175-8239-JBN-2024-0002en (PMC11420934; doi:10.1590/2175-8239-JBN-2024-0002en)
Supplement: Supplementary file 11 [file 2175-8239-jbn-46-4-e20240002-suppl6.pdf]

Braz. J. Nephrol. (J. Bras. Nefrol.)

https://doi.org/10.1590/2175-8239-JBN-2024-0002en

Supplementary Material to "Effect of urinary tract infection on the outcome of the allograft in patients with kidney transplantation"

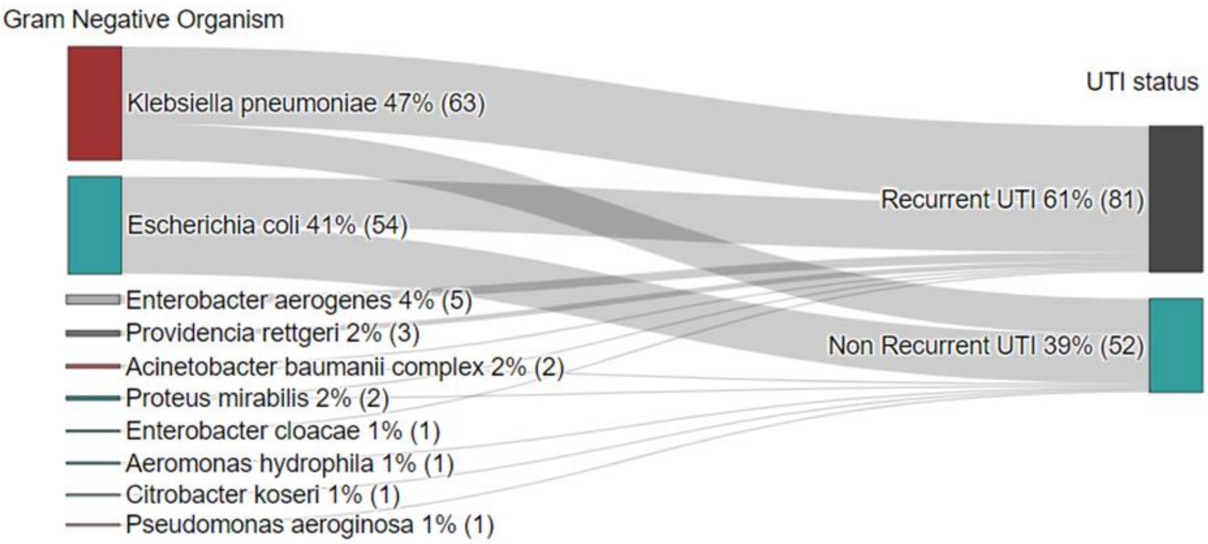

Figure S6. Sankey chart showing the antimicrobial profile of Gram-negative organisms causing post-kidney transplant UTI.
