# Supplementary material for: Effect of urinary tract infection on the outcome of the allograft in patients with kidney transplantation
Source: J Bras Nefrol. 2024 Sep 20;46(4):e20240002. doi: 10.1590/2175-8239-JBN-2024-0002en (PMC11420934; doi:10.1590/2175-8239-JBN-2024-0002en)
Supplement: Supplementary file 12 [file 2175-8239-jbn-46-4-e20240002-suppl13.pdf]

*Braz. J. Nephrol. (J. Bras. Nefrol.)*

https://doi.org/10.1590/2175-8239-JBN-2024-0002en

# Supplementary Material to "Effect of urinary tract infection on the outcome of the allograft in patients with kidney transplantation"

**Table S7.** Patient analysis of Gram-negative organisms with antibiotic resistance causing non-recurrent and recurrent post-kidney transplant UTIs (on case-by-case basis).

| | **Organism** | **Patient-level analysis of antimicrobial resistance patterns for Gram-negative organisms** | | | | | | | | **MDR** |
|---|---|---|---|---|---|---|---|---|---|---|
| | | **3rd Gen Cephalosporin** | **ESBL** | **4th Gen Cephalosporin** | **Carbapenem** | **Aminoglycoside** | **Fluoroquinolone** | **Nitrofurantoin** | **Cotrimoxazole** | |
| **Non-Recurrent UTI** | ***Acinetobacter baumanii* complex** | 1 | 0 | 1 | 1 | 1 | 1 | 0 | 1 | 1 |
| | ***Aeromonas*** | 0 | 0 | 0 | 1 | 0 | 0 | 0 | 0 | 0 |
| | ***Citrobacter koseri*** | 0 | 0 | 0 | 0 | 0 | 0 | 0 | 0 | 0 |
| | ***Escherichia coli*** | 22 | 8 | 19 | 8 | 14 | 23 | 6 | 20 | 18 |

| | Organism | Patient-level analysis of antimicrobial resistance patterns for Gram-negative organisms | | | | | | | | MDR |
|---|---|---|---|---|---|---|---|---|---|---|
| | | 3rd Gen Cephalosporin | ESBL | 4th Gen Cephalosporin | Carbapenem | Aminoglycoside | Fluoroquinolone | Nitrofurantoin | Cotrimoxazole | |
| | ***Klebsiella pneumoniae*** | 19 | 1 | 19 | 16 | 17 | 19 | 18 | 18 | 18 |
| | ***Pseudomonas aeroginosa*** | 1 | 0 | 1 | 0 | 0 | 1 | 0 | 0 | 1 |
| **Recurrent UTI** | ***Acinetobacter*** | 1 | 0 | 0 | 0 | 0 | 1 | 1 | 1 | 1 |
| | ***Enterobacter aerogenes*** | 0 | 0 | 0 | 0 | 0 | 3 | 5 | 0 | 0 |
| | ***Enterobacter cloacae*** | 1 | 1 | 0 | 0 | 0 | 1 | 1 | 1 | 1 |
| | ***Escherichia coli*** | 21 | 9 | 14 | 12 | 5 | 21 | 9 | 14 | 11 |
| | ***Klebsiella pneumoniae*** | 41 | 1 | 36 | 40 | 35 | 43 | 39 | 43 | 41 |
| | ***Proteus mirabilis*** | 1 | 0 | 1 | 1 | 0 | 2 | 2 | 1 | 1 |

| | Organism | Patient-level analysis of antimicrobial resistance patterns for Gram-negative organisms | | | | | | | | MDR |
|---|---|---|---|---|---|---|---|---|---|---|
| | | 3rd Gen Cephalosporin | ESBL | 4th Gen Cephalosporin | Carbapenem | Aminoglycoside | Fluoroquinolone | Nitrofurantoin | Cotrimoxazole | |
| | ***Providencia rettgeri*** | 3 | 0 | 3 | 3 | 3 | 3 | 3 | 3 | 3 |
