# Supplementary material for: Effect of urinary tract infection on the outcome of the allograft in patients with kidney transplantation
Source: J Bras Nefrol. 2024 Sep 20;46(4):e20240002. doi: 10.1590/2175-8239-JBN-2024-0002en (PMC11420934; doi:10.1590/2175-8239-JBN-2024-0002en)
Supplement: Translation

# Translation: Efeito da infecção do trato urinário no desfecho do aloenxerto em pacientes com transplante renal

**DOI:** 10.1590/2175-8239-JBN-2024-0002pt

**Authors:** Rahul Sai Gangula, Mahesh Eshwarappa, R Rajashekar, Hamsa Reddy, Pooja Prakash Prabhu, Gireesh M Siddaiah, Gurudev Konana Chennabasappa, Lia Sara Anish, Yousuff Mohammad

**Affiliations:** 1 Ramaiah University of Applied Sciences (RUAS), M. S. Ramaiah Medical College, Department of Nephrology, Bengaluru, Karnataka, India. Ramaiah University of Applied Sciences (RUAS) M. S. Ramaiah Medical College Department of Nephrology Bengaluru Karnataka India

**Keywords:** Infecção do Trato Urinário, ITU Recorrente, ITU Não Recorrente, Transplante

## Abstract

**Histórico:**

Infecções do trato urinário (ITU) são a segunda causa mais comum de disfunção do enxerto, responsáveis por morbidade significativa, sendo associadas à baixa sobrevida do enxerto e paciente. O objetivo deste estudo foi avaliar associação entre ITU pós-transplante renal e desfechos do enxerto.

**Métodos:**

Examinamos o efeito das ITU nos desfechos do enxerto em pacientes submetidos ao transplante renal entre janeiro/2010 e dezembro/2022. A população do estudo incluiu 349 transplantes renais, dos quais 74 apresentaram 140 eventos de ITU. Baseado no número de episódios de ITU, categorizamos os pacientes em três grupos.

**Resultados:**

Dos 349 receptores, 275 (74,4%) não apresentaram ITU, 47 (18,8%) apresentaram ITU não recorrentes (ITU-NR) e 27 (6,8%) apresentaram ITU recorrentes (ITU-R). O método de Kaplan-Meier mostrou que o status da ITU pós-TR foi um fator significativo na sobrevida do enxerto, sobrevida do enxerto censurada para óbito e sobrevida do paciente após acompanhamento de 5 anos (log rank, P < 0,001). ITU-R foram associadas à sobrevida muito baixa do enxerto e sobrevida do paciente comparadas com nenhuma ITU após acompanhamento de 5 anos (razão de risco [RR], 1,506; IC 95%, 1,233–1,840; P < 0,001 & RR, 1,529; IC 95%, 1,227–1,905; P = 0,001). ITU-R apresentaram maior probabilidade de serem associadas a organismos Gram-negativos multirresistentes (*Klebsiella pneumonia* ou *Escherichia coli*) resistentes à nitrofurantoína (RR, 2,753; IC 95%, 1,257–6,032; P = 0,01) e carbapenem (RR, 2,064; IC 95%, 0,988–4,314; P = 0,05).

**Conclusão:**

Em comparação com ausência de ITU, ITU-R foram associadas a piores desfechos do enxerto e paciente após acompanhamento de 5 anos, enquanto ITU-NR foram associadas a piores desfechos do enxerto e paciente em longo prazo.

## Introdução

A doença renal em estágio terminal é o estágio final da doença renal crônica, em que os rins não conseguem mais suprir as necessidades do corpo^1^. Embora diversas modalidades de terapias renais substitutivas estejam disponíveis, o transplante renal garante maior longevidade com a melhor qualidade de vida, além de ser o método de melhor custo-benefício^1^. Dessa forma, o transplante renal é atualmente considerado a melhor modalidade de terapia renal substitutiva^1^.

Depois de doenças cardiovasculares, as infecções são a segunda causa mais comum de mortalidade em receptores de transplante renal^1^. As infecções do trato urinário (ITU) estão entre as infecções mais comuns após o transplante renal e podem levar à disfunção do enxerto e comprometer a função do rim transplantado^1,2^. Embora as ITU possam ocorrer a qualquer momento após o transplante renal, elas são mais comuns no primeiro ano pós-transplante e podem levar à sepse, rejeição celular aguda, função prejudicada do aloenxerto, perda do enxerto e óbito do paciente^1–3^. Assim, a maioria dos centros prescreve pelo menos de 3 a 6 meses de profilaxia antimicrobiana após o transplante renal, embora os regimes possam variar^1–4^. Apesar da administração rotineira de profilaxia antimicrobiana durante o período inicial pós-transplante, as infecções representam uma significativa morbidade e mortalidade em receptores de transplante^4^.

A incidência de ITU em receptores de transplante renal é altamente variável entre os estudos (7% a 80%). Essa variação se deve a diferenças nas populações de pacientes, desenhos dos estudos e definições de ITU. Algumas ITU podem ser assintomáticas ou causar somente sintomas leves, enquanto outras podem levar a complicações graves que afetam a função do enxerto. Aproximadamente 19% dos pacientes desenvolvem pielonefrite aguda nos primeiros 2 anos após o transplante renal. Os fatores de risco comuns incluem cateteres de longa permanência e stents duplo J após o transplante, descontinuação precoce de antibióticos, comprimento reduzido do ureter transplantado, ausência de esfíncter entre o ureter transplantado e a bexiga nativa e dose total elevada de imunossupressão imediatamente após o transplante^5–7^. Os fatores de risco para recorrência incluem idade avançada, doador do sexo feminino, doador falecido, bexiga neurogênica, histórico de ITU pré-operatória e episódios de rejeição aguda (tratados com esteroides ou regimes imunossupressores)^5–7^. Foi relatada a ocorrência de ITU recorrentes em 4–72% dos receptores de transplante^8^.

Embora a maioria dos estudos^8–12^ tenha demonstrado uma associação entre ITU pós-transplante renal e deterioração da função do enxerto, o impacto da ITU nos desfechos de longo prazo do enxerto e do paciente é menos claro e apresenta resultados divergentes. Este estudo foi organizado com os seguintes objetivos para abordar essas lacunas críticas de conhecimento:

1) Determinar a associação entre ITU pós-transplante renal e os desfechos do enxerto.
2) Determinar a associação entre ITU pós-transplante renal e a sobrevida do paciente.
3) Descrever e comparar os perfis de resistência microbiológica e antimicrobiana de pacientes com ITU não recorrente (ITU-NR) e ITU recorrente (ITU-R).

## Métodos

### Desenho do Estudo e População de Pacientes

Esse estudo de coorte, observacional e de base hospitalar foi realizado em um hospital de cuidados terciários em Bengaluru, Karnataka, Índia. O estudo foi aprovado pelo Comitê de Ética Institucional (DRP/IFP1085/2023). Foram incluídos todos os pacientes submetidos a transplante renal em nosso instituto entre janeiro de 2010 e dezembro de 2022.

### Definições e Agrupamento de Eventos de ITU

A ITU foi definida como a presença de quaisquer dos sintomas clínicos de febre, disúria, ardência ao urinar, dor abdominal ou na região lombar, urina com odor fétido, aumento da frequência de micção e uma amostra de urocultura revelando crescimento de um único microrganismo com > 10^5^ unidades formadoras de colônias bacterianas por mL.

Todos os pacientes foram categorizados em 3 grupos com base em seu status de ITU após o transplante renal (sem ITU, ITU não recorrente e ITU recorrente). A ITU recorrente foi definida como um paciente que apresentou 2 ou mais episódios de ITU em qualquer período de 6 meses (ou) 3 ou mais episódios em qualquer período de 12 meses durante o acompanhamento pós-transplante. A ITU não recorrente foi definida como todos os pacientes com histórico de ITU após o transplante renal que não foram classificados no grupo ITU recorrente. Ausência de ITU foi definida como todos os pacientes que nunca apresentaram nenhum episódio de ITU durante o acompanhamento pós-transplante renal.

A resistência a antibióticos foi definida de acordo com as diretrizes do *Clinical and Laboratory Standards Institute* (CLSI) para testes de suscetibilidade a antimicrobianos. A multirresistência foi definida como a não suscetibilidade a pelo menos um agente em três ou mais categorias diferentes de antimicrobianos.

A falência do enxerto foi definida como o comprometimento do funcionamento do rim enxertado no receptor, com necessidade de terapia renal substitutiva por mais de 3 meses.

A bacteriúria assintomática foi definida como pacientes que não apresentaram sintomas sugestivos de ITU, mas cuja cultura de urina mostrou crescimento de organismos com >10^5^ unidades formadoras de colônias por mL.

A cistite aguda foi definida como uma infecção do trato urinário confinada à bexiga em uma mulher saudável, na pré-menopausa e não gestante.

### Imunossupressão Pós-Transplante

Todos os pacientes foram estratificados de acordo com a necessidade de indução, baseada no tempo de diálise, incompatibilidades de HLA, compatibilidade de grupo sanguíneo, idade do receptor e idade do doador. Todos os pacientes de alto risco receberam ATG, enquanto os pacientes de risco moderado receberam basiliximabe. Os pacientes de baixo risco não receberam ATG ou basiliximabe. Além disso, todos os pacientes receberam esteroides parenterais no dia do transplante e 2 dias após o transplante. Todos os receptores de transplante foram submetidos à anastomose ureterovesical por meio da técnica de ‘Lich-Gregoir’.

Todos os pacientes receberam um regime imunossupressor de manutenção com três medicamentos, composto por Tacrolimus, Micofenolato de Mofetila e Prednisolona. O tacrolimus foi iniciado com 0,08–0,1 mg/kg/dia e, posteriormente, a dose foi ajustada de acordo com os níveis mínimos de tacrolimus. Considerou-se um nível-alvo mínimo de tacrolimus de 9–10 ng/mL até 3 meses após o transplante, um alvo de 7–9 ng/mL de 3 a 12 meses após o transplante e um alvo de 5–7 ng/mL a partir de então. O micofenolato de mofetila 500 mg foi administrado duas a três vezes ao dia. Os esteroides foram reduzidos gradualmente para 10 mg 3 meses após o transplante e foram mantidos em 10 mg até 12 meses após o transplante. Posteriormente, os esteroides foram reduzidos gradualmente para 5–7,5 mg por dia, dose que foi mantida em seguida.

### Política de Cateter e Stent DJ

Um stent duplo J foi inserido em todos os receptores de aloenxertos durante o transplante como procedimento padrão e foi removido de forma asséptica entre 3 e 8 semanas após o transplante. Todos os receptores de transplante foram submetidos a inserção de cateteres de Foley durante o transplante renal. Em geral, eles foram removidos entre os dias 5 e 9 do pós-operatório, a menos que o paciente tivesse bexiga neurogênica ou qualquer outra indicação que recomendasse a cateterização prolongada com Foley.

### Quimioprofilaxia Pós-Transplante

Pacientes soronegativos para CMV que receberam rim de um doador soropositivo para CMV e receptores de transplante renal de doador falecido foram tratados com valganciclovir durante os 6 primeiros meses pós-transplante. Além disso, administrou-se trimetoprim-sulfametoxazol para *Pneumocystis jiroveci* durante os primeiros três meses pós-transplante, se tolerado.

### Bacteriúria Assintomática

Se um paciente apresentasse bacteriúria assintomática nos primeiros 2 meses após o transplante, a urocultura era repetida. Se o paciente apresentasse duas culturas de urina consecutivas que resultassem em >10^5^ unidades formadoras de colônias do mesmo patógeno, ele era tratado com antibióticos por 5 dias de acordo com a sensibilidade da cultura e considerado como ITU na análise. A bacteriúria assintomática após 2 meses pós-transplante não foi tratada com antibióticos e não foi considerada na análise.

### Tratamento da ITU

Todos os eventos de ITU sem sepse foram tratados empiricamente com cefalosporinas de terceira geração. Eventos de urossepsia foram tratados empiricamente com carbapenêmicos. Após os relatórios de cultura de urina, a prescrição foi ajustada e antibióticos adequados foram administrados por um período ideal (ou seja, 14 a 21 dias). Todos os pacientes com ITU recorrente foram submetidos a exames clínicos, laboratoriais e de imagem para identificar a causa e foram tratados da maneira adequada.

### Profilaxia Antibiótica Pós-ITU

Os receptores de transplante com ITU recorrentes foram tratados adequadamente e a profilaxia antibiótica foi administrada por 6 semanas a 3 meses.

### Diagnóstico de Rejeição, Nefropatia por Vírus BK

Os episódios de rejeição foram diagnosticados por meio de biópsia do rim enxertado, avaliados de acordo com a classificação de Banff^13^ e tratados de acordo. A triagem por PCR para BKV foi realizada periodicamente em todos os receptores de transplante. A nefropatia viral por BK foi confirmada utilizando coloração SV40 no tecido da biópsia do aloenxerto.

### Desfechos

O desfecho primário foi a sobrevida global do enxerto em pacientes com ITU. Os desfechos secundários incluíram função anual do enxerto, sobrevida do enxerto censurada para óbito, sobrevida do paciente e associação entre o status da ITU e a resistência a antibióticos. Os pacientes que tinham enxertos funcionantes no dia do óbito foram censurados para analisar a sobrevida do enxerto censurada para óbito.

### Coleta de Dados

Os dados foram coletados usando um proforma pré-delineado e, posteriormente, inseridos no Microsoft Office Professional Plus Excel 2016, versão 16.0 (Microsoft Corp, Redmond, EUA). Para evitar possíveis erros, a entrada de dados foi verificada em dois níveis (entrada no proforma e entrada do proforma na planilha do Excel) por dois observadores independentes.

### Análise Estatística

Foram realizadas estatísticas descritivas para variáveis categóricas e contínuas. O teste de normalidade de Shapiro-Wilk foi utilizado para verificar a distribuição dos dados. Um valor de P < 0,05 no teste de normalidade de Shapiro-Wilk foi considerado significativo e a distribuição foi classificada como distribuição não-Gaussiana. Variáveis quantitativas com distribuição gaussiana foram resumidas como médias e desvios padrão. As variáveis quantitativas, que apresentaram distribuição assimétrica, foram resumidas como medianas e intervalos interquartis. Todos os pacientes foram categorizados em três grupos (sem ITU, ITU não recorrente, ITU recorrente) com base na ITU pós-transplante renal. As características dos receptores de transplante renal, dos doadores e do transplante foram comparadas usando o teste qui-quadrado para dados categóricos e a análise de variância (ANOVA) unidirecional ou o teste H de Kruskal-Wallis para variáveis contínuas. Os desfechos de sobrevida do paciente, do enxerto e do enxerto censurada para óbito foram calculados e comparados por meio de análises de sobrevida de Kaplan-Meier (teste log-rank, P < 0,05 considerado significativo) e regressão univariada de Cox. Os tempos médio e mediano de sobrevida com intervalos de confiança de 95% foram calculados para os desfechos do enxerto e do paciente.

Os modelos de risco proporcional de Cox foram estimados usando variáveis selecionadas por meio da abordagem *backward stepwise*. Variáveis associadas à falência do enxerto (P < 0,20) foram consideradas para inclusão no modelo. Somente as variáveis que alteraram significativamente a relação entre o status da ITU pós-transplante e o desfecho, resultando em uma alteração ≥ 10% na RR associada, foram incluídas no modelo multivariado final. A ITU foi considerada uma variável dependente do tempo. As razões de risco não ajustadas para resistência a antibióticos foram comparadas com o status da ITU para espécies Gram-negativas em cada episódio e no nível do paciente. Um valor de P < 0,05 foi considerado estatisticamente significativo. Todas as análises estatísticas foram realizadas utilizando o *Statistical Package for the Social Sciences (SPSS) Statistics*, versão 25 (IBM Co., Armonk, NY, EUA), e a calculadora DATA *tab* Statistics (Graz, Áustria).

## Resultados

Um total de 349 pacientes foram submetidos a transplante renal durante o período especificado (2010–2022) e todos os pacientes foram incluídos na análise final (Figura 1). A duração média do acompanhamento foi de 70 meses (IIQ 31,5–104 meses). Um total de 287 pacientes eram do sexo masculino e 62 do sexo feminino (Figura S1). A média de idade dos receptores de transplante foi de 37,49 anos, com um desvio padrão de 11,92 anos. Durante o período do estudo, 74 pacientes apresentaram 140 episódios de ITU. Desses, 47 (13%) pacientes apresentaram ITU-NR, enquanto 27 (8%) pacientes tiveram ITU-R. Um total de 56 (40%) e 84 (60%) episódios de ITU foram registrados nos pacientes com ITU-NR e ITU-R, respectivamente. As outras características basais estão resumidas na Tabela 1.

**Figura 1:** Fluxograma basal do desenho do estudo.

**Tabela 1:** Comparação de características relacionadas ao receptor, doador e transplante com base no status da ITU pós-transplante renal

Características		Total (n = 349)	Sem ITU (n = 275)	ITU Não Recorrente (n = 47)	ITU Recorrente (n = 27)	Valor de P
**Características dos receptores**						
**Idade**	**Média**	37,49	36,29	40,83	42,74	**0,003**
	**Desvio Padrão**	11,92	11,66	12,03	12,16	
**Sexo**	**Masculino**	287 (82,2%)	228 (82,9%)	36 (76,6%)	23 (85,2%)	0,530
	**Feminino**	62 (17,8%)	47 (17,9%)	11 (23,4%)	4 (14,8%)	
**Doença Básica**	**GNC**	201 (57,6%)	171 (62,1%)	21 (44,7%)	9 (33,3%)	**0,002**
	**DRD**	66 (18,9%)	49 (17,8%)	11 (23,4%)	6 (22,2%)	0,601
	**NIC**	53 (15,2%	36 (13,1%)	12 (25,5%)	5 (18,5%)	0,079
	**Outras**	10 (2,9%)	7 (2,5%)	0 (0,0%)	3 (11,1%)	**0,017**
	**Desconhecida**	19 (5,4%)	12 (4,4%)	3 (6,4%)	4 (14,8%)	0,070
**Diabetes Mellitus**		59 (16,9%)	45 (16,4%)	8 (17,0%)	6 (22,2%)	0,742
**Hipertensão**		281 (80,5%)	216 (78,5%)	38 (80,1%)	21 (77,8%)	0,668
**Doença Cardíaca Isquêmica**		29 (8,3%)	27 (9,8%)	1 (2,1%)	1 (3,7%)	0,141
**Doença Cerebrovascular**		9 (2,6%)	4 (14,5%)	2 (4,2%)	3 (11,1%)	**0,008**
**Transtorno Convulsivo**		13 (3,7%)	9 (3,3%)	4 (8,5%)	0 (0,0%)	0,123
**Hipotireoidismo**		39 (11,2%)	32 (11,6%)	5 (10,6%)	2 (7,4%)	0,796
**ITU Pré-Transplante**		12 (3,4%)	8 (2,9%)	2 (4,2%)	2 (7,4%)	0,450
**Características dos doadores**						
**Idade**	**Média**	45,26	44,77	46,15	46,30	0,638
	**Desvio Padrão**	10,80	11,46	11,54	12,29	
**Sexo**	**Masculino**	94 (26,9%)	68 (24,7%)	17 (36,2%)	9 (33,3%)	0,194
	**Feminino**	255 (73,1%)	209 (75,3%)	28 (63,8%)	18 (66,7%)	
**Doador falecido**		46 (13,2%)	31 (11,3%)	10 (21,3%)	5 (18,5%)	0,086
**Doador vivo marginal**		44 (12,6%)	38 (13,8%)	4 (8,5%)	2 (7,4%)	**<0,001**
**Características do transplante**						
**Incompatibilidade HLA > 3**		145 (41,5%)	111 (40,4%)	20 (42,6%)	14 (51,9%)	0,509
**Segundo Transplante**		8 (2,3%)	5 (1,8%)	2 (4,3%)	1 (2,1%)	0,518
**Profilaxia com TMP-SMX**		297 (85,1%)	241 (87,6%)	36 (76,6%)	20 (74,1%)	**0,036**

### Características dos Receptores

Os receptores com média de idade mais elevada (Figura S2) tinham maior probabilidade de apresentar ITU-NR e ITU-R do que pacientes sem eventos de ITU (p = 0,039; p = 0,019, respectivamente), e não houve diferença estatisticamente significativa entre ITU-NR e ITU-R (p = 0,779). Os receptores com ITU-R apresentaram maior probabilidade de ter doença cerebrovascular do que os pacientes sem eventos de ITU (P = 0,007). No entanto, não houve diferença estatisticamente significativa entre ITU-NR e pacientes sem eventos de ITU (P = 0,498). Receptores de transplante renal com glomerulonefrite crônica e CAKUT como doença renal nativa (Tabela 1, Figura S3) foram associados a ITU-R (vs. Sem ITU, P = 0,019; vs. Sem ITU e ITU-NR, P = 0,009 e P = 0,029, respectivamente).

### Características dos Doadores

Os receptores de transplante renal com doadores vivos marginais foram associados a ITU-R e ITU-NR quando comparados com pacientes sem episódios de ITU (P = 0,007; p = 0,003, respectivamente).

### Características do Transplante

Receptores de transplante com diabetes mellitus pós-transplante (DMPT) foram associados a ITU-R em comparação com pacientes com ITU-NR e pacientes sem episódios de ITU (P = 0,005; P = 0,002, respectivamente). Os pacientes que não receberam profilaxia com trimetoprim-sulfametoxazol desenvolveram ITU-NR (vs. Sem ITU, P = 0,049). Receptores de transplante renal com função tardia do enxerto apresentaram ITU não recorrente (vs. Sem ITU, P = 0,038), mas a diferença não foi estatisticamente significativa para ITU recorrente (vs. Sem ITU, P = 0,459). Pacientes com stents DJ persistentes por > 4 semanas apresentaram ITU-R (vs. Sem ITU, P < 0,001). Os receptores de transplante que não receberam nenhum agente de indução foram associados à ITU-R (P = 0,019) em comparação com os pacientes que não apresentaram nenhuma infecção do trato urinário.

### Número de Episódios de ITU em Cada Paciente

Um total de 41 (55%) pacientes apresentou somente 1 evento de ITU, 16 (22%) pacientes apresentaram 2 eventos de ITU, 10 (13%) pacientes apresentaram uma soma de 3 eventos de ITU e 7 (10%) pacientes apresentaram mais de 3 eventos de ITU (Figura S4).

### Tempo Mediano Para Evento de ITU

O tempo mediano desde o transplante até a ITU inicial em ITU-NR e ITU-R foi de 41 dias (IIQ 16,0 – 624,5 dias) e 26 dias (IIQ 19,5 – 56,5 dias), respectivamente (Figura S5).

### Desfechos do Enxerto e do Paciente no Acompanhamento de 5 Anos

A análise de sobrevida de Kaplan-Meier mostrou que o status de ITU pós-TR foi significativamente diferente na sobrevida do enxerto, na sobrevida do enxerto censurada para óbito e na sobrevida do paciente após acompanhamento de 5 anos (Figuras 2–4, Tabelas S1–S3).

**Figura 2:** Comparação da sobrevida do enxerto por status de ITU após transplante renal (acompanhamento por 5 anos). Os resultados são mostrados para receptores sem ITU, com ITU não recorrente e com ITU recorrente.

**Figura 3:** Comparação da sobrevida do enxerto censurada para óbito por status de ITU após transplante renal (acompanhamento por 5 anos). Os resultados são mostrados para receptores sem ITU, com ITU não recorrente e com ITU recorrente.

**Figura 4:** Comparação da sobrevida do paciente por status de ITU após transplante renal (acompanhamento por 5 anos). Os resultados são mostrados para receptores sem ITU, com ITU não recorrente e com ITU recorrente.

Após ajuste para fatores de confusão na análise de regressão multivariada de Cox, a ITU-NR [RR 2,318; p = 0,001] foi associada a um risco maior de falência do enxerto em comparação com ausência de ITU ou ITU-R (Tabelas 2 e 3).

**Tabela 2:** Modelo de regressão de Cox de fatores dos receptores associados à falência global do enxerto entre os receptores de transplante renal

Parâmetro		Razão de risco ajustada	Intervalo de Confiança de 95%	Valor de P
**Status da ITU**	**Sem ITU**	Comparador	–	–
	**ITU Não Recorrente**	2,318	1,414–3,800	**0,001**
	**ITU Recorrente**	1,528	0,657–3,551	0,325
**Idade do Receptor**		1,026	1,007–1,046	**0,008**
**Sexo do Receptor**		0,657	0,403–1,070	0,091
**Hipertensão**		1,182	0,956–1,343	0,065
**Diabetes**		1,304	0,969–1,756	0,080
**Diabetes Pós-Transplante**		1,912	1,423–2,257	**0,007**
**Transplante anterior**		1,113	0,550–2,254	0,766

**Tabela 3:** Modelo de regressão de Cox de fatores do transplante e do doador associados à falência global do enxerto entre receptores de transplante renal

Parâmetro		Razão de Risco Ajustada	Intervalo de Confiança de 95%	Valor de P
**Status da ITU**	**Sem ITU**	Comparador	–	–
	**ITU Não Recorrente**	2,318	1,414–3,800	**0,001**
	**ITU Recorrente**	1,528	0,657–3,551	0,325
**Função Tardia do Enxerto**		2,202	1,614–3,003	**<0,001**
**Incompatibilidade HLA > 3**		1,307	1,041–1,641	**0,021**
**Agente de indução**	**ATG**	Comparador	–	–
	**Basiliximabe**	2,467	1,437–4,236	0,001
	**Nulo**	1,138	0,724–1,790	0,003
**Rejeição/Glomerulopatia de transplante**		8,840	5,472–14,281	**<0,001**
**Recidiva de doença básica**		5,452	3,117–9,537	**<0,001**
**Nefropatia por vírus BK**		1,496	0,986–1,984	**0,032**
**Sexo do Doador**		1,004	0,615–1,637	0,989
**Doador Vivo Marginal**		1,598	1,262–2,023	**<0,001**
**Doador Falecido**		1,961	1,420–2,709	**<0,001**

Um total de 34 (10%) pacientes apresentaram perda do enxerto durante o período de observação, dos quais 26 (9%) não tiveram ITU e 8 (17%) tiveram ITU não recorrente. O tempo mediano de sobrevida global do enxerto foi de 72 meses (IC 95% 64,654–79,346). No entanto, as medianas dos tempos de sobrevida global do enxerto para os grupos sem ITU, ITU-NR e ITU-R foram de 84 meses (IC 95% 73,763–94,237), 60 meses (IC 95% 48,817–71,183) e 36 meses (IC 95% 1,271–70,729), respectivamente (Tabelas S4 e S5).

Um total de 56 (16%) pacientes foram a óbito durante o período de observação, dos quais 37 (13%) não apresentaram ITU, 13 (27%) apresentaram ITU não recorrente e 6 (22%) apresentaram ITU recorrente. O tempo mediano de sobrevida dos pacientes do grupo ITU-NR foi de 118 meses (IC 95% 90,932–145,068) (Tabela S6).

### Perfil Microbiológico e Padrões de Resistência

Um total de 140 eventos de ITU foram registrados em 74 pacientes durante o período de observação, dos quais 133 (95%) foram devido a bactérias Gram-negativas e 7 (5%) a bactérias Gram-positivas (Tabela 4). As bactérias Gram-negativas mais comuns foram *Klebsiella pneumoniae* (47%) e *Escherichia coli* (41%), enquanto as bactérias Gram-positivas mais comuns foram as espécies de Enterococcus (Figura S6). A maioria dos organismos eram multirresistentes, sendo responsáveis por 38 (73,07%) e 58 (71,6%) eventos em ITU não recorrentes e recorrentes, respectivamente (Tabela S7).

**Tabela 4:** Análise em nível de paciente dos padrões de resistência antimicrobiana para organismos gram-negativos causadores de ITU pós-transplante renal recorrentes e não recorrentes

Resistente a antibióticos	ITU Não Recorrente (52)	ITU Recorrente (81)	RR	IC 95%	Valor de P
**Cefalosporina de 3^a^ Geração**	43 (87,8)	68 (85)	0,791	0,276–2,264	0,081
**Beta-lactâmico de espectro estendido**	9 (22,5)	11 (16,0)	0,709	0,270–1,856	0,486
**Cefalosporina de 4^a^ Geração**	40 (81,6)	54 (67,5)	0,467	0,197–1,106	0,664
**Aminoglicosídeos**	32 (64)	43 (53,7)	0,654	0,316–1,351	0,253
**Fluoroquinolones**	45 (90)	73 (91,2)	1,159	0,347–3,871	0,812
**Nitrofurantoína**	25 (54,3)	59 (76,6)	2,753	1,257–6,032	**0,010**
**Carbapenem**	26 (53,0)	56 (70,0)	2,064	0,988–4,314	**0,049**
**Cotrimoxazol**	39 (81,2)	63 (78,7)	0,855	0,347–2,108	0,736

## Discussão

Embora não existam critérios únicos para distinguir ITU precoces de tardias, as ITU que ocorrem dentro de um ano após o transplante foram denominadas ITU precoces em diversos estudos^1–7^. O momento dos episódios de ITU pós-transplante é fundamental, uma vez que estudos indicam que a ITU precoce é um fator de risco para o desenvolvimento de sepse e rejeição do aloenxerto^4–7^. De forma semelhante, a ITU recorrente tardia aumenta a probabilidade de disfunção do aloenxerto e perda do enxerto^8^. Entretanto, esses efeitos não foram consistentes em todas as pesquisas^4,8,10^. Dados recentes sugerem que até mesmo um único episódio de ITU pode comprometer o desempenho do aloenxerto em longo prazo em receptores de transplante renal^4,12^.

Os processos de dano ao aloenxerto por patógenos que têm como alvo o trato urinário estão ligados à resposta inflamatória associada à ITU contra a invasão bacteriana, que é causada pela desregulação imunológica e pela ativação local e sistêmica de citocinas como TNF-α, IL-1, IL-6 e IL-8^7^. Além disso, acredita-se que a liberação de citocinas desempenhe um papel na etiologia da rejeição do aloenxerto ao acelerar a exposição dos tecidos do aloenxerto aos HLAs, resultando na ativação de leucócitos e células endoteliais vasculares^7^. Em determinadas situações, o desenvolvimento de pielonefrite aguda pode potencialmente levar à insuficiência crônica do aloenxerto devido à injúria renal direta^12^.

A infecção por organismos uropatogênicos Gram-negativos virulentos com estruturas especializadas, como fímbrias P (pili associado à pielonefrite), está fortemente ligada à disfunção aguda do aloenxerto^7^. Ataques repetidos esgotam a capacidade regenerativa do tecido do enxerto e promovem fibrose irreversível^7^. Como resultado, em receptores de transplante com ITU recorrente tardia, o dano ao aloenxerto pode resultar na formação de inúmeras anormalidades cicatriciais localizadas que podem ser observadas por tomografia computadorizada por emissão de fóton único com ácido di-mercaptossuccínico marcado com Tecnécio Tc 99m (99mTc-DMSA SPECT)^7^.

O presente estudo é único por considerar todos os pacientes que foram submetidos a transplante renal durante o período do estudo, sem exclusão de nenhum. O objetivo deste estudo foi avaliar e determinar a associação entre ITU após transplante renal e desfechos do enxerto e do paciente. Em conformidade com outros estudos^4,8^, a maioria dos casos de eventos de ITU (tanto ITU-R quanto ITU-NR) ocorreram dentro de um ano após o transplante. Isso pode ser devido à maior dose líquida de imunossupressão imediatamente após o transplante.

De acordo com os achados do presente estudo, os receptores mais velhos, os receptores de doadores vivos marginais e os receptores de transplante que desenvolveram FTE ou DMPT apresentaram maior probabilidade de desenvolver ITU. Isso pode ser explicado por um equilíbrio imunológico alterado, níveis persistentemente elevados de glicose no sangue e função de barreira da mucosa prejudicada durante essas condições especiais.

Um novo achado do presente estudo foi que a ITU-NR esteve associada a um maior risco de falência do enxerto e desfechos inferiores tanto para o paciente quanto para o enxerto. Isso contradiz a maioria dos estudos anteriores^4,8^ que mostraram que as ITU-R estão geralmente associadas a piores desfechos do paciente e do enxerto. Embora o atual estudo tenha demonstrado um aumento na mortalidade entre os pacientes com ITU-R, esse aumento não foi estatisticamente significativo quando comparado com pacientes sem ITU. Uma possível explicação para a menor sobrevida do enxerto entre ITU-NR em comparação com ITU-R pode ser o fato de pacientes com ITU-R terem recebido tratamento empírico com carbapenêmicos, profilaxia antibiótica prolongada e monitoramento e acompanhamento rigorosos. Isso pode ser devido à abordagem intensiva da análise da causa raiz de ITU recorrentes e estratégias de tratamento precoce. Alguns pacientes apresentaram refluxo para o rim nativo e foram submetidos a procedimentos cirúrgicos (1 paciente foi submetido a nefrectomia do rim nativo e 3 receptores de transplante tiveram um agente de volume de dextranômero/ácido hialurônico injetado na junção vesicoureteral). No entanto, a discrepância pode ser atribuída ao tempo de acompanhamento mais curto nesses casos, uma vez que a maioria dos pacientes com ITU-R havia sido submetida a transplante recentemente (nos últimos 5 anos). Isso fica evidente quando uma análise de sobrevida é realizada com um acompanhamento de apenas 5 anos.

A Índia é um país grande e diversificado, com uma ampla gama de doenças infecciosas devido à densidade populacional, ao saneamento inadequado, à desigualdade econômica, ao acesso à saúde e aos diferentes níveis de cobertura vacinal. Devido ao aumento associado de morbidade e mortalidade, a resistência antimicrobiana se tornou um grave problema de saúde. Semelhante a estudos anteriores, os organismos Gram-negativos foram responsáveis pela maioria das ITU, com *Klebsiella pneumoniae* e *Escherichia coli* representando aproximadamente 47% e 41%, respectivamente. Os patógenos causadores de ITU-R foram análogos aos envolvidos em ITU-NR, sem diferenças estatisticamente significativas observadas entre os dois grupos para qualquer organismo.

Quando analisadas em nível de paciente e evento de ITU, tanto as ITU-NR quanto as ITU-R tinham maior probabilidade de serem causadas por organismos multirresistentes. A resistência aos carbapenêmicos ou à nitrofurantoína está associada ao desenvolvimento de ITU recorrentes, levando a desafios no tratamento. Isso provavelmente se deve à prática ineficaz do manejo de antibióticos. Tal situação está levando a um aumento de patógenos multirresistentes, especialmente resistência à nitrofurantoína e ao carbapenem, uma vez que esses antibióticos são atualmente utilizados com regularidade no tratamento de ITU complicadas.

Os desfechos divergentes observados no presente estudo podem ser explicados pelas técnicas de tratamento precoce e pela abordagem de análise da causa raiz utilizadas na avaliação das ITU-R. A desvantagem significativa do estudo atual é que a maioria dos pacientes com ITU-R foram submetidos a transplantes recentes; portanto, não foi possível estudar seu desfecho em longo prazo (>5 anos). No entanto, planeja-se acompanhar todos esses pacientes recentemente transplantados em longo prazo e investigar os desfechos do transplante.

A inclusão de todos os receptores de transplante renal ao longo do período da pesquisa e o extenso período de acompanhamento foram os pontos fortes deste estudo. Uma análise para os fatores de risco independentes para disfunção do enxerto relacionada à ITU foi realizada após ajuste para variáveis de confusão, por meio de uma análise multivariada de regressão proporcional de Cox. Para quantificar o risco independente de fatores de confusão, foram obtidos riscos proporcionais e razões de risco. A bacteriúria assintomática ocorrida nos primeiros 2 meses após o transplante foi confirmada por cultura repetida e tratada se o mesmo organismo fosse isolado^14^.

No entanto, este estudo apresentou diversas limitações. Não avaliamos a prevalência global de bacteriúria assintomática e sua relação com o surgimento de ITU após o transplante renal. Como este foi um estudo de centro único, não é possível extrapolar os achados para toda a população-alvo. No presente estudo, pacientes do sexo feminino foram sub-representadas como receptoras de transplante e as doadoras foram fortemente sobrerepresentadas. Isso pode ser devido ao fato de que, na Índia, os doadores são predominantemente mães ou cônjuges^15^. Portanto, os resultados não podem ser generalizados para outros centros de transplante com uma distribuição equilibrada de receptores e doadores por sexo. Este estudo não explorou a relação entre os diferentes tipos e durações da terapia e seu impacto nos desfechos. O grupo ITU-R do presente estudo foi submetido ao transplante mais recentemente e não teve um período de acompanhamento longo. Essa é uma das limitações do estudo que poderia ser abordada por um acompanhamento mais prolongado desses receptores de transplante.

Em resumo, constatamos que o grupo ITU-NR apresentou desempenho do enxerto, sobrevida do enxerto e desfechos do paciente consistentemente piores do que o grupo sem ITU. O momento mais provável para ITU após o transplante renal é dentro de um ano após o transplante, e a causa mais comum são as bactérias Gram-negativas multirresistentes. Além disso, constatou-se que determinados fatores estavam independentemente associados à falência do enxerto, incluindo receptores do sexo masculino, diabetes mellitus pós-transplante, glomerulopatia do enxerto, recidiva de doença subjacente/doença glomerular *de novo*, doadores vivos marginais, doadores falecidos, função tardia do enxerto, mais de 3 incompatibilidades HLA, nefropatia por vírus BK, episódios de rejeição, ausência de indução e basiliximabe como agente de indução.

## Conclusões

Em comparação com a ausência de ITU, as ITU-R foram associadas a piores desfechos do enxerto e do paciente após um acompanhamento de 5 anos, enquanto as ITU-NR foram associadas a piores desfechos do enxerto e do paciente em longo prazo. Dada a escassez de tratamentos disponíveis, é crucial combater a multirresistência a antimicrobianos, especialmente a resistência a carbapenêmicos. Para pessoas que receberam um transplante renal, até mesmo uma única ITU pode resultar em piores desfechos de longo prazo para o enxerto e para o paciente.

## Material Suplementar

O seguinte material online está disponível para o presente artigo:

Figure S1 – Sankey chart showing the gender of kidney transplant recipients and donors.

Figure S2 – Box and whisker plot with data points representing the age of kidney transplant recipients and donors.

Figure S3 – Sankey chart showing the native kidney disease of kidney transplant recipients.

Figure S4 – Sankey chart showing the total number of UTI episodes in each patient.

Figure S5 – Vertical raincloud plot showing the duration of the first episode of UTI post-kidney transplant in non-recurrent and recurrent UTI groups.

Figure S6 – Sankey chart showing the antimicrobial profile of Gram-negative organisms causing post-renal transplant UTI.

Table S1 – Mean and median survival time of graft survival at a 5 years follow-up.

Table S2 – Mean and median survival time of death-censored graft survival at a 5 years follow-up.

Table S3 – Mean and median survival time of patient survival at a 5 years follow-up.

Table S4 – Mean and median survival time of overall graft survival.

Table S5 – Mean and median survival time of overall death-censored graft survival.

Table S6 – Mean and median survival time of overall patient survival.

Table S7 – Patient analysis of Gram-negative organisms with antibiotic resistance causing non-recurrent and recurrent post-kidney transplant UTIs (on case-by-case basis).
